# Multimodal Diagnostic Ultrasound for Congestion, Perfusion, and Ultrafiltration Tolerance in Maintenance Hemodialysis: A Narrative Review

**DOI:** 10.3390/diagnostics16162678

**Published:** 2026-08-21

**Authors:** Kexin Yin, Jie Sun, Kun Liu

**Affiliations:** 1 Department of Ultrasound Medicine, The Third People’s Hospital of Hubei Province Graduate Joint Training Base, School of Medicine, Wuhan University of Science and Technology, Wuhan 430033, China; ykx772453174@163.com; 2 Department of Ultrasound Medicine, Tongji Hospital, Tongji Medical College, Huazhong University of Science and Technology, Wuhan 430030, China; 3 Department of Ultrasound Medicine, Third People’s Hospital of Hubei Province, Wuhan 430033, China

**Keywords:** hemodialysis, diagnostic ultrasound, lung ultrasound, ultrafiltration tolerance, venous congestion, artificial intelligence

## Abstract

Background: Maintenance hemodialysis is characterized by repetitive changes in fluid distribution, blood pressure, and organ perfusion. Conventional clinical examination, empirical dry-weight adjustment, and biomarkers do not fully resolve the compartment-specific nature of congestion in this population. This narrative review reframes ultrasound-based volume assessment as a multimodal diagnostic problem involving pulmonary congestion, intravascular filling, systemic venous congestion, cardiac reserve, tissue response, and perfusion vulnerability. Methods: We synthesized clinically relevant evidence indexed in PubMed and Google Scholar for studies published between January 2016 and April 2026, prioritizing dialysis-specific randomized trials, prospective cohorts, systematic reviews, consensus statements, and methodological studies related to diagnostic ultrasound, Doppler-based congestion assessment, contrast-enhanced ultrasound, elastography, artificial intelligence, point-of-care ultrasound, and remote ultrasound monitoring. Results: Lung ultrasound currently has the strongest dialysis-specific evidence for detecting and tracking pulmonary congestion. Inferior vena cava ultrasound provides adjunctive information on intravascular filling and right-sided pressure but is not a surrogate for total body water. Echocardiographic parameters help characterize filling pressure and cardiac tolerance to fluid removal, whereas venous Doppler and the Venous Excess Ultrasound Score provide an emerging approach to systemic venous congestion. Elastography and contrast-enhanced ultrasound remain investigational tools for tissue characterization and perfusion vulnerability, while AI-assisted analysis, handheld point-of-care ultrasound, and tele-ultrasound may improve standardization, automated B-line quantification, and scalability. Conclusions: Different ultrasound modalities answer different diagnostic questions in maintenance hemodialysis. A compartment-specific framework integrating congestion, perfusion, and cardiac-reserve domains may better support individualized ultrafiltration planning, hemodynamic risk assessment, and future outcome-oriented research.

## 1. Introduction

Chronic kidney disease (CKD) is a major global public health challenge, and hemodialysis remains the most widely used renal replacement therapy worldwide [1,2,3]. In patients undergoing maintenance hemodialysis, volume status management is both a cornerstone of treatment and a persistent clinical challenge [4]. Persistent volume overload contributes to hypertension, left ventricular hypertrophy, and heart failure, whereas excessive ultrafiltration may cause volume depletion, intradialytic hypotension, muscle cramps, syncope, and reduced dialysis tolerance [5,6,7]. Therefore, accurate and dynamic assessment of volume status is essential for optimizing dialysis management and improving cardiovascular outcomes.

Traditional assessment tools fall short of clinical needs owing to their single-dimensional constraints. While the multicenter randomized controlled LUST trial confirmed that lung ultrasound-guided volume management markedly alleviates pulmonary congestion, it yielded no significant reduction in all-cause mortality or composite cardiovascular hard endpoints [8]. This paradox underscores the intrinsic limitation of single-chamber assessments centered solely on pulmonary congestion. In hemodialysis patients, volume dysregulation reflects a multifaceted pathophysiological state involving altered multi-chamber fluid distribution alongside compromised cardiac reserve. Therefore, constructing an integrated, multidimensional framework for volume assessment is imperative—forming the central premise of this review.

Unlike prior reviews that focused on a checklist-like enumeration of assessment tools under the implicit assumption that all parameters reflect total body water, this review begins with an examination of the body fluid compartments and their pathophysiological mechanisms and provides a systematic overview and supplementation focusing on the following five aspects.

① Introducing a compartment-specific evaluation perspective: Precisely mapping distinct ultrasound parameters to their respective fluid compartments (intravascular, interstitial, and pulmonary) to delineate their specific diagnostic targets; ② Dissecting discordant ultrasound phenotypes: Elucidating the mechanisms underlying clinically common paradoxical findings (e.g., IVCD in the absence of pulmonary B-lines) and their implications for ultrafiltration tolerance and hemodynamic risk; ③ Constructing an integrated interpretive framework: Providing a clinical decision-making framework to reconcile conflicting ultrasound results, thereby informing individualized ultrafiltration strategies and guiding future research; ④ Tracing technological translation: Tracking the translational trajectory of ultrasound applications from general patient populations to specialized hemodialysis settings, while charting potential pathways forward; ⑤ Offering a prospective outlook on AI: Evaluating the clinical translation, future potential, and current limitations of artificial intelligence in this field.

## 2. Literature Search and Selection Strategy

This narrative review synthesizes relevant evidence from a diagnostic ultrasound perspective. A comprehensive literature search was conducted across PubMed (National Library of Medicine, Bethesda, MD, USA) and Google Scholar (Google LLC, Mountain View, CA, USA) for studies published between January 2016 and April 2026, using combinations of the following terms: “hemodialysis”, “volume status”, “fluid overload”, “lung ultrasound”, “B-lines”, “inferior vena cava”, “echocardiography”, “VExUS”, “bioimpedance”, “ultrasound elastography”, “contrast-enhanced ultrasound”, “Doppler ultrasound”, “point-of-care ultrasound”, “artificial intelligence”, and “remote ultrasound”. Additionally, highly relevant landmark and classic publications published prior to 2016 were manually identified. Screening was performed by one reviewer and verified by a second reviewer.

Study Selection and Eligibility Criteria Studies were eligible if they evaluated ultrasound or related modalities for assessing volume status, pulmonary congestion, intravascular filling, venous congestion, cardiac function, tissue characteristics, organ perfusion, or ultrafiltration tolerance in patients undergoing maintenance hemodialysis (MHD). Priority was given to clinical trials, randomized controlled trials, prospective cohort studies, systematic reviews, consensus statements, and clinically relevant methodological studies involving the MHD population. Evidence from non-dialysis populations was included only when dialysis-specific data were insufficient.

Studies were excluded if they: (1) were unrelated to volume assessment; (2) focused exclusively on pediatric or peritoneal dialysis populations; (3) lacked sufficient clinically relevant detail; or (4) were published solely as conference abstracts. When multiple studies reported similar findings, priority was given to representative, recent, and methodologically robust studies, while seminal landmark publications were retained where appropriate. The literature identification and selection process is summarized in the supplementary flow diagram (Figure S1).

## 3. Narrative Evidence Synthesis and Classification

Regarding evidence maturity, evidence is qualitatively categorized into three tiers—mature, emerging, and exploratory—based on the volume and consistency of dialysis-specific studies, degree of clinical validation and standardization, and clinical applicability. Specifically, mature evidence indicates a robust and consistent evidence base within dialysis; emerging evidence points to a growing but limited body of literature and persistent heterogeneity; and exploratory evidence comprises preliminary, indirect, mechanistic, or feasibility data insufficient to support routine clinical application. Notably, this classification serves as a descriptive narrative assessment rather than a formal evidence-grading system.

Furthermore, the current clinical positioning of each technology is determined through a holistic evaluation incorporating evidence maturity, validation in dialysis populations, implementation feasibility, degree of standardization, clinical interpretability, and relevance to diagnostic ultrasound practice.

The use of generative AI-assisted tools is disclosed in the Acknowledgments Section in accordance with MDPI reporting recommendations.

## 4. Conventional Approaches to Volume Assessment and Their Diagnostic Limitations

### 4.1. Clinical Assessment

Clinical assessment primarily includes blood pressure, heart rate, peripheral edema, jugular venous distension, and pulmonary crackles. However, these signs lack specificity and sensitivity [9], and their presence often indicates that volume overload has already reached a relatively advanced stage [10]. Peripheral edema may be influenced by hypoproteinemia, impaired venous return, or local vascular disease [11], while pulmonary crackles are a late manifestation of pulmonary edema and may be absent in early pulmonary congestion [12].

### 4.2. Dry-Weight Assessment

Dry-weight adjustment is often based on interdialytic weight gain, symptoms, blood pressure, and ultrafiltration tolerance. This empirical trial-and-error approach is subjective and may lead to repeated shifts between overhydration and volume depletion, compromising dialysis adequacy and tolerance.

### 4.3. Biomarkers

Biomarkers such as B-type natriuretic peptide and N-terminal pro-B-type natriuretic peptide reflect ventricular wall stress and may indirectly indicate volume burden [13]. However, baseline levels are frequently elevated in hemodialysis patients because clearance is affected by kidney function and dialysis modality [14], and these markers are influenced by heart failure, diastolic dysfunction, and myocardial hypertrophy. Their specificity as isolated indicators of volume overload is therefore limited [15].

## 5. Multimodal Diagnostic Ultrasound for Compartment-Specific Assessment in Dialysis Patients

Diagnostic ultrasound provides a non-invasive, repeatable, and bedside approach for assessing volume-related physiological changes in maintenance hemodialysis. Its clinical value depends on recognizing that each modality interrogates a different compartment and answers a different diagnostic question. Intravascular filling, pulmonary congestion, systemic venous congestion, cardiac reserve, tissue edema, and organ perfusion may be concordant in some patients but discordant in others. This region-specific interpretation is central to the practical application of multimodal ultrasound in dialysis care.

### 5.1. Intravascular Filling: Inferior Vena Cava Ultrasound

Early ultrasound-based volume assessment focused mainly on inferior vena cava diameter (IVCD) and the IVC collapsibility index. Because IVC size varies with right atrial pressure and intravascular filling, it has long been used as a bedside marker of intravascular volume. In hemodialysis, IVC-related parameters have been used to monitor volume changes, assess ultrafiltration adequacy, and guide dry-weight adjustment [16,17].

However, IVC measurements are affected by intra-abdominal pressure, respiratory variation, right-sided cardiac dysfunction, tricuspid regurgitation, and pulmonary hypertension [18,19]. In addition, the IVC is not a regular cylindrical structure and may move out of plane during respiration, introducing measurement error and reducing reproducibility [20]. Therefore, IVC ultrasound provides dialysis-specific information on intravascular filling and ultrafiltration response, but it should be interpreted as an adjunctive dynamic parameter rather than a stand-alone surrogate of total body water [21,22,23].

### 5.2. Pulmonary Congestion and Extravascular Lung Water: Lung Ultrasound

Lung ultrasound (LUS) semi-quantitatively assesses extravascular lung water by detecting B-lines, which arise from increased pulmonary interstitial fluid and correlate with pulmonary congestion [24,25]. The 28-zone scanning protocol remains a widely used reference approach, although abbreviated protocols have also been evaluated in dialysis populations [26,27].

LUS has accumulated the strongest dialysis-specific evidence base among ultrasound tools for congestion assessment. Predialysis LUS scores correlate with bioimpedance-defined overhydration, chest radiographic congestion, and natriuretic peptides, and they decrease after fluid removal [28,29]. In a large prospective cohort of maintenance hemodialysis patients, pulmonary congestion assessed by LUS independently predicted all-cause mortality and cardiovascular events [30].

A 2025 systematic review showed that diagnostic studies and randomized trials support the feasibility of B-line monitoring in dialysis, but also highlighted substantial heterogeneity and overall risk of bias, with no consistent proof of benefit for long-term hard outcomes [31]. More recently, a multicenter randomized trial in 100 hemodialysis patients using a simplified 8-zone protocol showed that LUS-guided dry-weight adjustment reduced B-line burden and pulmonary congestion over 60 days without increasing intradialytic hypotension [32]. Abbreviated protocols may enhance implementation, but the 28-zone approach remains the reference when precise congestion quantification is required [33]. Accordingly, LUS should presently be framed as the most clinically usable dialysis-specific ultrasound adjunct for detecting and tracking pulmonary congestion, rather than as a stand-alone surrogate for whole-body volume status.

Methodologically, B-line quantification varies across scanning protocols, including 28-, 14-, and 8-zone approaches, limiting comparability between studies [26,27]. Image acquisition and interpretation may also be affected by obesity, chronic lung disease, atelectasis, pleural adhesions, and examination timing [34,35,36]. Importantly, B-lines are not specific to volume overload. In hemodialysis patients, volume-related B-lines are usually bilateral, diffuse, relatively symmetric, and decrease after ultrafiltration, whereas non-volume-related abnormalities are more often associated with pleural irregularity, patchy distribution, or subpleural lesions [37,38].

### 5.3. Cardiac Reserve and Filling Pressure: Echocardiographic Parameters

Cardiac ultrasound complements volume assessment by evaluating cardiac tolerance to fluid removal. In hemodialysis, the key question is not only whether excess fluid is present, but also whether it can be safely removed. Traditional parameters such as E/e′, tricuspid regurgitation velocity, and left atrial volume estimate filling pressure, but their sensitivity to acute dialysis-related volume changes is inconsistent [39].

Left atrial strain may integrate left atrial compliance and chronic filling-pressure burden, and recent data suggest that left atrial reservoir and conduit strain respond to hemodialysis-related preload changes [40,41]. Similarly, global longitudinal strain can detect subclinical myocardial dysfunction despite preserved left ventricular ejection fraction and may help identify patients at risk for adverse cardiovascular outcomes [42]. Afterload-corrected indices such as pressure-strain loop-derived myocardial work may better reflect myocardial energy expenditure during blood pressure fluctuations, but dialysis-specific evidence remains preliminary [43,44,45,46].

### 5.4. Systemic Venous Congestion and Organ Venous Outflow: VExUS and Venous Doppler Assessment

**Figure 1:**
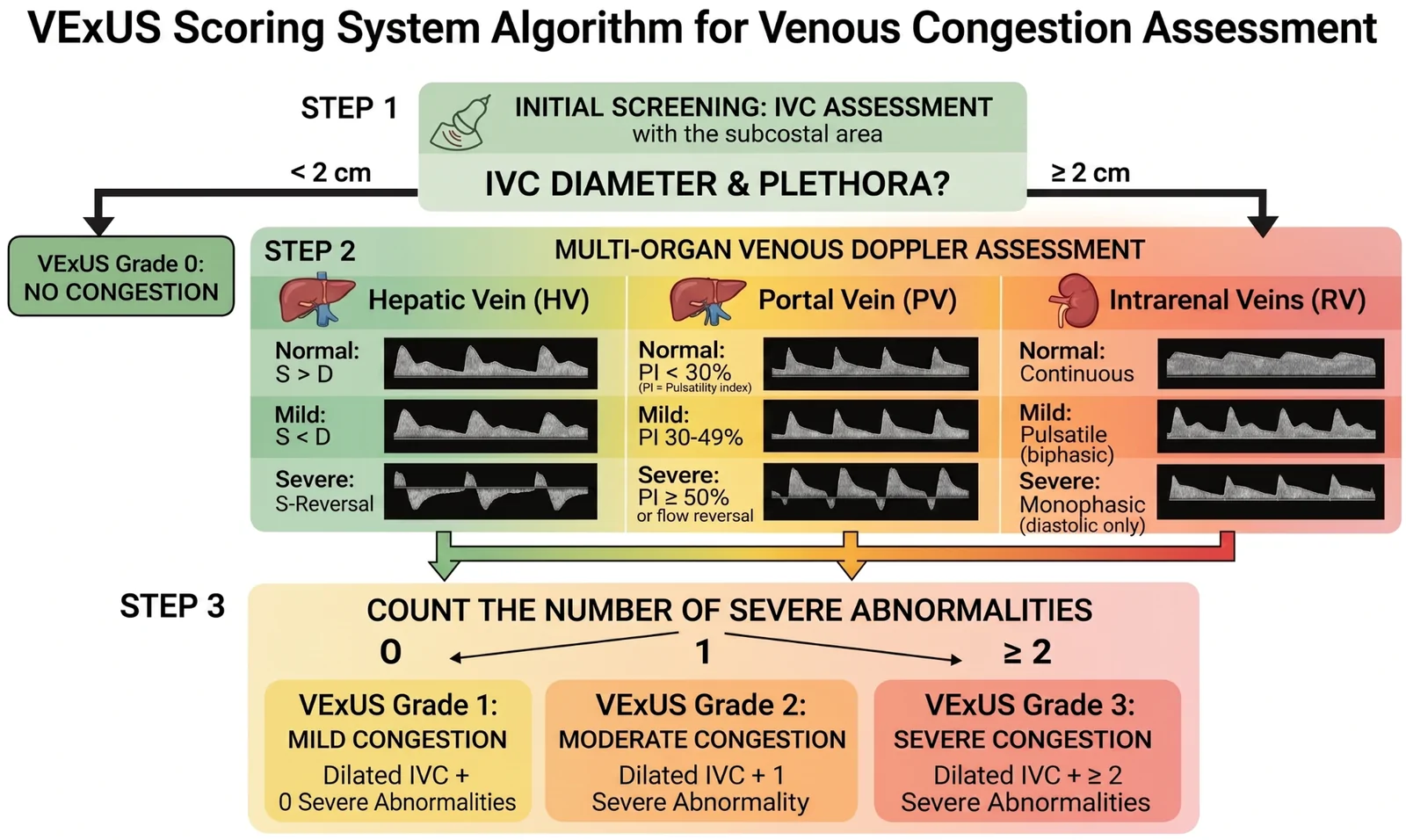
VExUS (Venous Excess Ultrasound) scoring system algorithm for venous congestion assessment. The algorithm consists of three sequential steps. Step 1 involves initial evaluation of the inferior vena cava (IVC) diameter; an IVC < 2 cm indicates no congestion (VExUS Grade 0), whereas an IVC ≥ 2 cm requires further venous Doppler evaluation. Step 2 entails multi-organ venous Doppler assessment of the hepatic vein, portal vein, and intrarenal veins. Step 3 determines the final VExUS grade according to the number of severe Doppler abnormalities. Abbreviations: IVC, inferior vena cava; HV, hepatic vein; PV, portal vein; RV, renal vein.

The VExUS (Venous Excess Ultrasound Score) system combines IVC assessment with Doppler interrogation of the hepatic, portal, and intrarenal veins. It was developed to quantify systemic venous congestion and organ venous outflow abnormalities, thereby shifting the focus from total fluid volume alone to venous return, organ congestion, and perfusion pressure (Figure 1) [47,48]. Venous congestion may reduce organ perfusion pressure and contribute to interstitial edema, microcirculatory dysfunction, and organ injury, especially in cardiorenal syndromes [49,50].

Dialysis-specific evidence for VExUS remains limited but is evolving. In the 2024 ACUVEX study, only a minority of maintenance hemodialysis patients above target dry weight had an elevated VExUS score, and all improved during fluid removal; importantly, elevated scores clustered in patients with biventricular systolic dysfunction, raising the possibility that VExUS may reflect cardiorenal venous failure rather than simple excess body water alone [51]. A separate 2024 study in hospitalized patients with end-stage kidney disease showed that portal vein pulsatility changed more sensitively than IVC diameter during ultrafiltration, whereas hepatic venous Doppler was less responsive after a single session [52]. In 2026, a prospective observational study in hospitalized ESRD patients further showed that VExUS changed dynamically before and after hemodialysis and may capture hemodynamic change beyond IVC collapsibility alone [53]. Taken together, these findings support physiologic plausibility, but the evidence base remains small, predominantly observational, and enriched for selected or inpatient populations. VExUS should therefore be presented as an exploratory adjunct for systemic venous congestion and ultrafiltration tolerance, not yet as a validated routine guide to dry-weight prescription in maintenance hemodialysis.

## 6. Integrated Diagnostic Interpretation and Clinical Decision-Making

**Figure 2:**
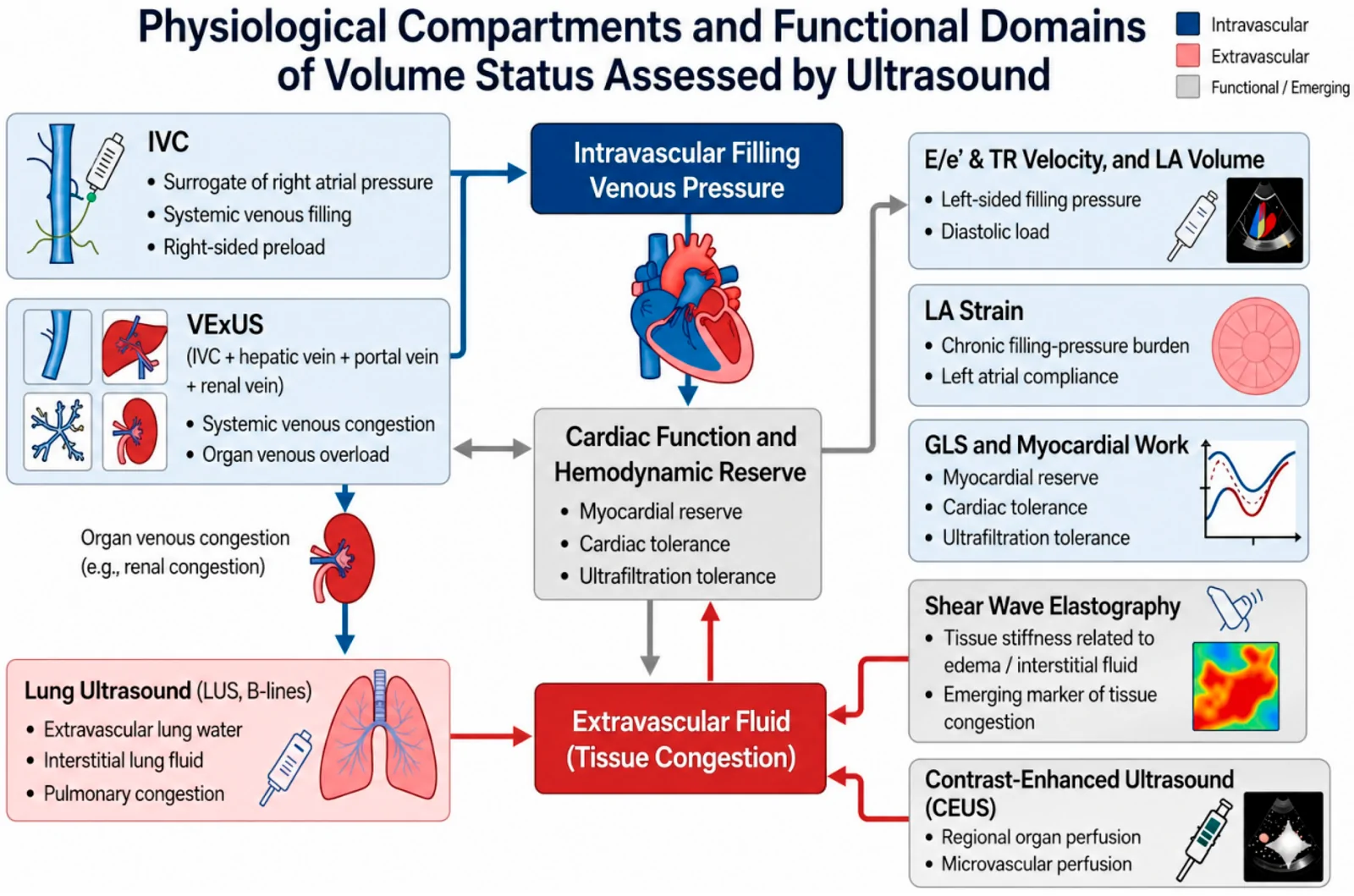
Compartment-specific framework for multimodal ultrasound-based volume assessment in maintenance hemodialysis. The figure summarizes how ultrasound-based techniques assess pulmonary congestion, intravascular filling, systemic venous congestion, cardiac reserve, tissue congestion, and organ perfusion. This framework emphasizes that dialysis volume assessment should move beyond single-parameter interpretation toward integrated evaluation of congestion, perfusion, cardiac reserve, blood pressure instability, and ultrafiltration tolerance.

Because no single method fully captures volume status or cardiorenal risk, multimodal diagnostic interpretation is increasingly emphasized. Combining parameters across intravascular filling, pulmonary congestion, systemic fluid distribution, venous congestion, cardiac reserve, and perfusion may reduce single-tool bias and improve individualized decision-making (Figure 2).

### 6.1. Combined Interpretation of LUS and IVC Ultrasound

Combining IVC ultrasound, which reflects intravascular filling and right-sided pressure, with LUS, which reflects pulmonary interstitial fluid, is one of the most common combined approaches in current practice. This combination can identify patients with pulmonary congestion but a normal IVC, or patients with a dilated IVC but few B-lines, thereby reducing misinterpretation caused by reliance on a single parameter [16,21,54]. Nevertheless, prospective studies must still determine whether predefined combined ultrasound pathways improve hard outcomes such as hospitalization, intradialytic hypotension, cardiovascular events, and survival.

### 6.2. Integration with Bioelectrical Impedance Analysis

Bioelectrical impedance analysis (BIA) estimates body fluid volume and composition by measuring tissue impedance and is widely used as an objective adjunct for volume assessment in dialysis [55]. However, BIA is not a gold standard for volume assessment; its interpretation may be influenced by body geometry and electrolyte disturbances, while its use in patients with cardiac implantable electronic devices has been reevaluated in recent years [56,57].

LUS and BIA often show similar trends at the population level, but individual-level agreement may be limited. Chang et al. reported only 62% agreement between post-dialysis LUS and BIA classifications in hospitalized maintenance hemodialysis patients, with a kappa value of 0.11 [58]. This discordance reflects their different physiological targets: LUS primarily detects pulmonary interstitial congestion, whereas BIA estimates systemic fluid distribution and extracellular water [59,60]. Prognostic studies also support complementarity: pulmonary B-line burden predicts mortality independently of bioimpedance parameters, while relative fluid overload by bioimpedance improves risk prediction beyond traditional and echocardiographic models [61,62]. Recent evidence on technology-guided dry-weight adjustment suggests potential clinical value, but also highlights that outcome benefits and adverse symptoms may vary across protocols and patient populations [63,64,65].

### 6.3. Clinical Interpretation of Discordant Ultrasound Findings

A practical advantage of multimodal ultrasound assessment is the ability to interpret discordant findings across different compartments. Increased B-lines with a normal IVC may suggest pulmonary congestion driven by elevated left-sided filling pressure or altered alveolar-capillary permeability rather than simple intravascular expansion. A dilated IVC with few B-lines may indicate right-sided pressure overload or systemic venous congestion without overt pulmonary congestion. Bioimpedance-defined overhydration with limited B-lines may reflect systemic fluid excess that has not translated into pulmonary edema. Conversely, elevated VExUS scores with poor ultrafiltration tolerance may indicate venous congestion and limited cardiac reserve. These patterns illustrate why ultrasound findings should be integrated according to the dominant physiological question rather than interpreted as interchangeable markers of total body water.

### 6.4. Diagnostic Implications for Ultrafiltration Tolerance and Hemodynamic Instability

For dialysis clinicians, the practical objective of multimodal ultrasound is not merely to estimate whether a patient is wet or dry, but to identify the dominant phenotype that determines ultrafiltration tolerance. A patient with pulmonary congestion may still experience intradialytic hypotension if cardiac reserve is limited or vascular refilling is inadequate. Conversely, a dilated IVC or elevated VExUS score may indicate right-sided pressure overload or systemic venous congestion rather than directly removable total body water. Integrating LUS, IVC, echocardiographic parameters, venous Doppler, BIA, symptoms, blood pressure trajectories, and ultrafiltration rate may therefore provide a more physiologically coherent basis for individualized ultrafiltration planning and future diagnostic algorithms.

## 7. Emerging Diagnostic Technologies and AI-Assisted Ultrasound

### 7.1. Tissue Characterization: Ultrasound Elastography

Ultrasound elastography measures tissue stiffness and has been widely used to assess fibrosis in organs such as the liver and kidney [66]. Because tissue edema may alter stiffness, elastography could theoretically provide indirect information on tissue congestion. However, dialysis-specific evidence remains scarce; existing studies have mainly evaluated complications or tissue characteristics rather than volume status directly [67,68,69,70]. Its role in volume assessment therefore remains exploratory.

### 7.2. Contrast-Enhanced Ultrasound and Perfusion-Oriented Diagnostic Methodologies

Currently, contrast-enhanced ultrasound remains a hypothesis-generating rather than clinically validated modality for routine volume assessment in maintenance hemodialysis patients. The most relevant contemporary evidence in the dialysis context derives from a 2025 prospective CEUS study involving incident hemodialysis patients with preserved residual kidney function, which demonstrated an acute intradialytic reduction in renal perfusion correlating with ultrafiltration rate [71]. While mechanistically significant, this finding does not establish CEUS as a routine dry-weight assessment tool in prevalent maintenance hemodialysis populations. A more robust interpretation suggests that perfusion-oriented ultrasound techniques may facilitate phenotypic characterization of ischemic vulnerability and end-organ ultrafiltration intolerance in future investigations, particularly among patients with recurrent intradialytic hypotension or accelerated residual kidney function decline [72].

### 7.3. AI-Assisted, Handheld, and Home-Based Lung Ultrasound

While lung ultrasound (LUS) is widely used to evaluate pulmonary congestion in hemodialysis, its clinical utility is constrained by operator experience, protocol heterogeneity, variable image quality, and subjective interpretation thresholds [73,74]. AI-assisted LUS addresses these limitations through real-time acquisition guidance, automated quality control, B-line recognition, and quantitative reporting.

Clinical evidence for AI-assisted LUS currently operates at two levels. In non-dialysis settings, AI guidance enables non-expert healthcare professionals to perform diagnostic-quality 8-zone LUS with expert-level proficiency (98.3% success rate) [75]. In dialysis populations, early studies confirm the feasibility of AI-driven fluid overload detection [76], patient self-scanning with automated B-line counting [77], and home tele-ultrasound in high-risk elderly patients [78]. However, lacking prospective clinical outcome data, AI-assisted LUS remains an enabling technology rather than an established management tool.

Technologically, deep learning models have evolved from basic B-line counting and localization [79,80] to real-time multi-class artifact segmentation [81] and rapid object-detection frameworks (e.g., YOLO) [82]. Expanding beyond B-lines, recent paradigms incorporate pixel-wise spatial analysis [83], pleural effusion quantification [84], multi-feature LUS phenotyping [85], and standardized inferior vena cava (IVC) tracking [86]. Ultimately, the most clinically relevant future application of AI in hemodialysis may include the integration of LUS, IVC, VExUS, BIA, blood pressure, ultrafiltration rate, symptoms, and dialysis-machine data into interpretable decision-support models.

## 8. Limitations of Current Diagnostic Ultrasound Evidence

Although ultrasound-based volume assessment in hemodialysis has shown substantial clinical potential, current evidence still has important limitations. Most existing studies rely on cross-sectional analysis, small sample sizes, and single-center designs, with a relative lack of randomized controlled trials and long-term follow-up data. Consequently, the reproducibility, stability, and predictive value of relevant ultrasound indicators for hard endpoints have not yet been fully validated. Furthermore, there are no unified standards across studies regarding scanning protocols, measurement timepoints, parameter thresholds, and outcome definitions, which limits the comparability of results and, to some extent, hinders the standardized implementation of these techniques. Additionally, ultrasound assessment is highly operator-dependent and susceptible to individual patient factors.

Another important limitation is the mixture of dialysis-specific and extrapolated evidence. LUS and BIA have the most mature dialysis-specific evidence base, whereas VExUS, advanced echocardiographic strain parameters, elastography, CEUS, quantitative Doppler perfusion, AI-assisted ultrasound, and home tele-ultrasound remain supported mainly by feasibility, mechanistic, non-dialysis, or early observational data. This distinction should be made explicit when translating evidence into clinical applications.

## 9. Future Directions for Diagnostic Ultrasound in Dialysis Care

Future research should prioritize outcome-oriented trials of LUS-guided and multimodal ultrasound-guided diagnostic pathways, with predefined links between ultrasound findings, dry-weight adjustment, ultrafiltration rate, dialysis schedule modification, and cardioprotective therapy. Standardized protocols are needed for scan timing, body position, scanning zones, threshold definitions, reporting formats, and integration with dialysis-machine and blood pressure data.

Clinical validation of AI-assisted diagnostic ultrasound tools should be accelerated, but outcome benefit, external validity, workflow feasibility, and interpretability must be demonstrated before these tools can be considered established management strategies.

## 10. Conclusions

For patients undergoing maintenance hemodialysis, no single ultrasound technique can comprehensively assess fluid status or replace clinical judgment. Each technique has its own diagnostic target, strengths, and limitations (Table 1). Lung ultrasound, IVC ultrasound, echocardiographic parameters, VExUS, BIA, and emerging ultrasound-based technologies provide complementary information across pulmonary, intravascular, venous, cardiac, systemic fluid, tissue, and perfusion-related domains. Their evidence maturity and current clinical positioning are summarized in Table 2.

Therefore, volume assessment in hemodialysis should move from single-parameter interpretation toward multimodal, compartment-specific diagnostic integration. Future studies should determine whether ultrasound-guided strategies, particularly those supported by AI-assisted standardization, can improve clinically meaningful outcomes including intradialytic hypotension, hospitalization, cardiovascular events, survival, and quality of life.

## Figures and Tables

**Table 1:** Comparison of Core Applications, Advantages, and Limitations of Ultrasound-Based and Related Technologies for Volume Assessment in Hemodialysis Patients.

Technology	Target/Application	Main Advantages	Main Limitations
**IVC**	Intravascular filling/estimated right atrial pressure	• Non-invasive & repeatable • Guides clinical UF planning	• Affected by intra-abdominal pressure, respiration, and right-heart function; • Lacks dialysis-specific cutoffs
**LUS**	Pulmonary congestion/extravascular lung water	• High sensitivity for subclinical congestion • Enables dynamic monitoring & risk stratification	• B-lines are non-specific • Protocol variability & operator-dependent
**Cardiac US** **(E/e′, GLS, MW)**	Cardiac/Hemodynamics	• Identifies early subclinical dysfunction • Myocardial work reduces BP confounding	• Indirect fluid measure • Evidence remains limited in dialysis
**VExUS & Venous Doppler**	Systemic venous/organ outflow	• Phenotypes organ venous congestion • Evaluates organ congestion beyond total volume	• Technically demanding • Mostly observational dialysis evidence
**BIA**	Total body water/ECW	• Objective & highly repeatable • Reflects systemic fluid burden (complements LUS)	• Non-gold standard • Confounded by body geometry & electrolytes
**Elastography & CEUS**	Tissue edema/microvascular	• Quantifies tissue stiffness & microvascular changes • Tracks pre-/post-dialysis tissue edema	• Purely exploratory/hypothesis-generating • Unvalidated clinical endpoints
**AI & Home-based LUS**	Automated EVLW/telehealth	• Automated B-line scoring & QA • Reduces operator bias; enables remote tracking	• Unproven hard outcome benefits • Requires external algorithm validation

Note. IVC: inferior vena cava; UF: ultrafiltration; LUS: lung ultrasound; EVLW: extravascular lung water; US: ultrasound; E/e′: ratio of peak early transmitral flow velocity (E) to early diastolic mitral annular velocity (e′); LA strain: left atrial strain; GLS: global longitudinal strain; MW: myocardial work; BP: blood pressure; VExUS: Venous Excess Ultrasound Score; BIA: bioelectrical impedance analysis; ECW: extracellular water; CEUS: contrast-enhanced ultrasound; AI: artificial intelligence; QA: quality assurance.

**Table 2:** Evidence Maturity, Key Clinical Value, and Current Clinical Positioning of Technologies for Volume Assessment in Hemodialysis Patients.

Technology	Evidence Maturity in HD	Key Clinical Value	Clinical Positioning
**IVC**	Emerging [18,21]	Dynamic intravascular filling & UF response	Clinical adjunct
**LUS**	Mature [31,32]	Pulmonary congestion tracking & risk stratification	Clinical adjunct
**Traditional Echo** **(E/e′, TR, LAVI)**	Emerging [39]	LV filling pressure & cardiac risk stratification	Clinical adjunct
**LA Strain**	Exploratory [41]	Preload responsiveness & LA reserve assessment	Investigational adjunct
**GLS**	Exploratory [42]	Early subclinical LV dysfunction & risk profiling	Investigational adjunct
**Myocardial Work**	Exploratory [45,46]	Afterload-adjusted myocardial efficiency	Research only
**VExUS & Venous Doppler**	Emerging [51,52,53]	Organ venous congestion phenotyping & UF tolerance	Investigational adjunct
**BIA**	Mature [55,63,64]	Systemic fluid distribution & ECW tracking	Clinical adjunct
**US Elastography**	Exploratory [66,70]	Tissue stiffness & peripheral edema phenotyping	Research only
**CEUS & Perfusion US**	Exploratory [71,72]	Microvascular perfusion & UF ischemic risk	Research only
**AI-Assisted LUS**	Emerging [75,76]	Automated B-line scoring & acquisition QA	Investigational adjunct
**Home Tele-ultrasound**	Exploratory [77,78]	Remote pulmonary congestion tracking & care access	Research only

Note: Evidence maturity is qualitatively categorized into mature, emerging, and exploratory tiers based on the volume and consistency of dialysis-specific studies, degree of clinical validation and standardization, and clinical applicability; this represents a descriptive assessment rather than a formal evidence-grading framework. The clinical positioning of each technology is determined through a holistic evaluation incorporating evidence maturity, validation results in dialysis populations, implementation feasibility, degree of standardization, clinical interpretability, and relevance to diagnostic ultrasound practice.

## Data Availability

No new data were created or analyzed in this study. Data sharing is not applicable to this article.

## Supplementary Materials

The following supporting information can be downloaded at: https://www.mdpi.com/article/10.3390/diagnostics16162678/s1, Figure S1. Flow diagram of literature identification and selection for the narrative review. Studies published between January 2016 and April 2026 were identified through PubMed and Google Scholar and screened according to the predefined eligibility criteria. Additional seminal and classic studies published before 2016 were identified through citation searching. Eligible studies were included in the final narrative synthesis.

## Abbreviations

AI: artificial intelligence; BIA: bioelectrical impedance analysis; BNP: B-type natriuretic peptide; CEUS: contrast-enhanced ultrasound; CKD: chronic kidney disease; ESRD: end-stage renal disease; GLS: global longitudinal strain; IVC: inferior vena cava; IVCD: inferior vena cava diameter; LA strain: left atrial strain; LUS: lung ultrasound; MHD: maintenance hemodialysis; NT-proBNP: N-terminal pro-B-type natriuretic peptide; POCUS: point-of-care ultrasound; VExUS: Venous Excess Ultrasound Score.
