# Supplementary material for: Multimodal Diagnostic Ultrasound for Congestion, Perfusion, and Ultrafiltration Tolerance in Maintenance Hemodialysis: A Narrative Review
Source: Diagnostics (Basel). 2026 Aug 21;16(16):2678. doi: 10.3390/diagnostics16162678 (PMC13512204; doi:10.3390/diagnostics16162678)
Supplement: Supplementary file 1 [file diagnostics-16-02678-s001.zip › diagnostics-4371943-supplementary.pdf]

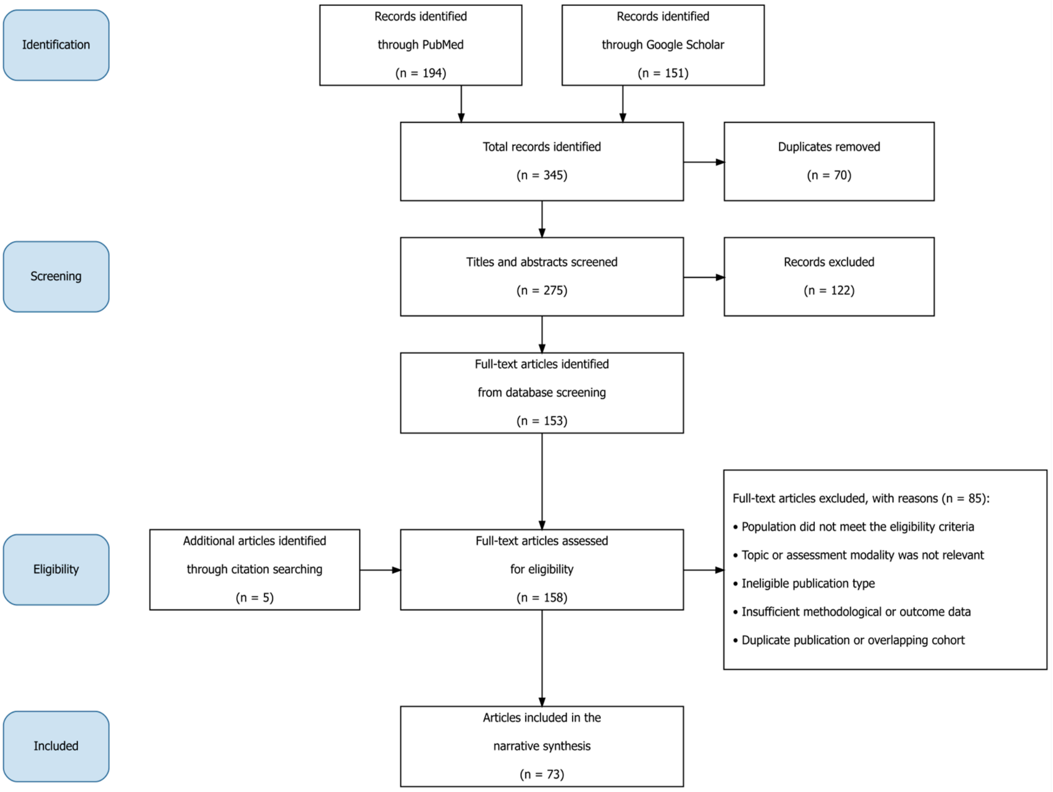

Supplementary Figure S1

Figure S1. Flow diagram of literature identification and selection for the narrative review. Studies published between January 2016 and April 2026 were identified through PubMed and Google Scholar and screened according to the predefined eligibility criteria. Additional seminal and classic studies published before 2016 were identified through citation searching. Eligible studies were included in the final narrative synthesis.
